# Evaluation of pharmacokinetic metrics and pharmacodynamic biomarkers associated with endoscopic healing in pediatric Crohn’s disease patients treated with anti-tumor necrosis factor biologics

**DOI:** 10.1093/crocol/otag088

**Published:** 2026-07-27

**Authors:** Courtney Bartel, Tomoyuki Mizuno, Rebekah Karns, Kei Irie, Kimberly Jackson, Kimberly Lynch, Abigail Samuels, Jeffrey S Hyams, Brendan Boyle, Jacob A Kurowski, Shehzad Saeed, Joshua D Noe, Kelly Chun, Jane Yang, Lee A Denson, Phillip Minar

**Affiliations:** Department of Pediatrics, Division of Gastroenterology, Hepatology, and Nutrition, Cincinnati Children’s Hospital Medical Center, Cincinnati, OH, United States; Department of Pediatrics, Division of Translational and Clinical Pharmacology, Cincinnati Children’s Hospital Medical Center, Cincinnati, OH, United States; Department of Pediatrics, University of Cincinnati College of Medicine, Cincinnati, OH, United States; Department of Pediatrics, Division of Gastroenterology, Hepatology, and Nutrition, Cincinnati Children’s Hospital Medical Center, Cincinnati, OH, United States; Department of Pediatrics, Division of Translational and Clinical Pharmacology, Cincinnati Children’s Hospital Medical Center, Cincinnati, OH, United States; Department of Pediatrics, Division of Gastroenterology, Hepatology, and Nutrition, Cincinnati Children’s Hospital Medical Center, Cincinnati, OH, United States; Department of Pediatrics, Division of Gastroenterology, Hepatology, and Nutrition, Cincinnati Children’s Hospital Medical Center, Cincinnati, OH, United States; Department of Internal Medicine, University of Cincinnati School of Medicine, Department of Veterans Affairs, Cincinnati, OH, United States; Department of Pediatrics, Division of Digestive Diseases, Hepatology and Nutrition, Connecticut Children’s Medical Center, Hartford, CT, United States; Department of Pediatrics, Division of Gastroenterology, Hepatology and Nutrition, Nationwide Children’s Hospital, Columbus, OH, United States; Department of Pediatrics, Division of Gastroenterology, Hepatology and Nutrition, Cleveland Clinic, Cleveland, OH, United States; Department of Pediatrics, Division of Gastroenterology, Hepatology and Nutrition, Wright State University and Dayton Children’s Hospital, Dayton, OH, United States; Department of Pediatrics, Division of Gastroenterology, Hepatology and Nutrition, Children’s Hospital of Wisconsin, Milwaukee, WI, United States; Esoterix, LabCorp Specialty Lab, Calabasas, CA, United States; Esoterix, LabCorp Specialty Lab, Calabasas, CA, United States; Department of Pediatrics, Division of Gastroenterology, Hepatology, and Nutrition, Cincinnati Children’s Hospital Medical Center, Cincinnati, OH, United States; Department of Pediatrics, University of Cincinnati College of Medicine, Cincinnati, OH, United States; Department of Pediatrics, Division of Gastroenterology, Hepatology, and Nutrition, Cincinnati Children’s Hospital Medical Center, Cincinnati, OH, United States; Department of Pediatrics, University of Cincinnati College of Medicine, Cincinnati, OH, United States

**Keywords:** infliximab clearance, neutrophil CD64, monocyte CD64

## Abstract

**Background:**

Crohn’s disease (CD) patients achieving deep remission, defined as clinical remission with endoscopic healing (EH), have a lower rate of CD-related adverse events. EH can be achieved with anti-tumor necrosis factor (TNF) dose optimizations directed by pharmacokinetic (PK) and pharmacodynamic (PD) biomarkers. The primary aim was to identify PK metrics and PD biomarker cut-points associated with EH.

**Methods:**

In this multicenter, cross-sectional study, we enrolled CD patients (age 1-22 years) who received an anti-TNF biologic (infliximab or adalimumab) for >6 months and underwent ileocolonoscopy. EH was defined as a simple endoscopic score-CD (SES-CD) < 3.

**Results:**

Eighty-seven patients were enrolled with EH found in 55.2%. Lower levels of novel biomarkers neutrophil CD64, monocyte CD64, and soluble CD64 were associated with EH with cut-points of <4.5 ratio (AUROC 0.76), <44.6 ratio (AUROC 0.67), and <15.9 ng/mL (AUROC 0.67), respectively. There was no difference in the mean trough concentrations of infliximab or adalimumab between EH and non-EH, but the median infliximab clearance was higher in non-EH (0.26 L/day) compared to EH (0.21 L/day, *P* = .02). In our infliximab multivariable model, slower infliximab clearance, lower platelet count, and higher serum albumin were strong predictors for EH (AUC 0.90).

**Conclusions:**

We identified novel PK and PD cut-points for biomarkers associated with EH, including infliximab clearance and the novel CD64 biomarkers. Furthermore, we developed a multivariable PK and PD model for EH that, following validation, could guide dose escalation (PK failures) or prompt a switch to an alternate advanced therapeutic for PD failures.

## Graphical Abstract

**Graphical Abstract:**
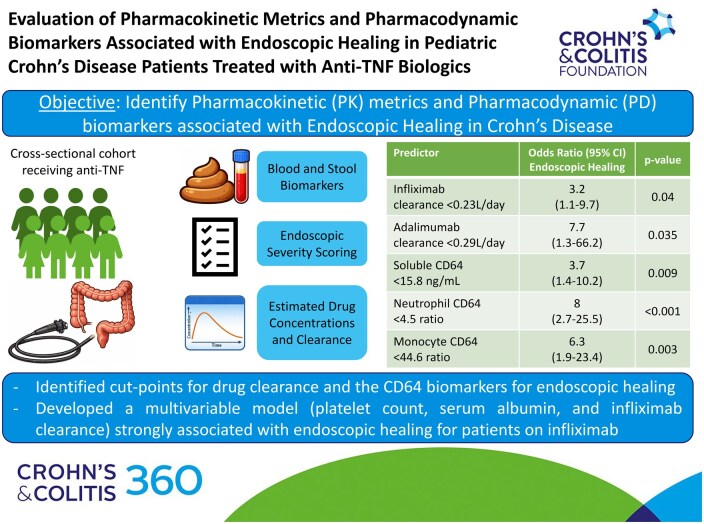

Key Messages *What is already known?*

Patients with Crohn’s disease (CD) who achieve both clinical remission and endoscopic healing (EH) (deep remission) have better long-term outcomes with fewer disease-related complications. Previous studies have evaluated pharmacokinetic (PK) metrics and pharmacodynamics (PD) biomarkers of EH

*What is new here?*

We identified routine and novel CD64 PD biomarkers and PK metrics with cut-points associated with EH specific to pediatrics. We also identified that anti- tumor necrosis factor drug clearance rate, but not absolute concentration, correlated with EH in our cohort of pediatric CD patients. Infliximab clearance, platelet count, and serum albumin were utilized to develop a multivariable regression model to predict EH.

*How can this study help patient care?*

By identifying cut-points for PD biomarkers and PK metrics associated with EH, clinicians can better guide therapy optimizations or consider a change in therapy when patients exhibit acceptable PK but demonstrate PD failure.

## Introduction

Crohn’s disease (CD) is a chronic illness of relapsing and remitting intestinal inflammation resulting from a profound immune dysregulation in genetically susceptible individuals.^1^ With the young age of onset, pediatric patients are susceptible to prolonged intestinal damage that may result in growth failure, intestinal strictures with possible bowel obstruction, and penetrating complications (fistula or abscess formation).^2^^,^^3^ Several advanced therapies are now approved for use in adult patients with CD. For children with CD, the tumor necrosis factor-alpha (TNF) inhibitors, infliximab, and adalimumab, are the only 2 biologic therapies currently approved by the United States Food and Drug Administration for moderate to severe CD with other advanced therapies often used off-label for children refractory to anti-TNF therapy.^4^^,^^5^

While standard (labeled) dosing regimens of infliximab and adalimumab are associated with improved early outcomes, the majority of patients will require dose intensification or shortening of the interval to maintain response secondary to rapid drug clearance.^6^^,^^7^ In fact, the leading causes of secondary nonresponse to anti-TNF include either low drug exposure and/or the development of neutralizing auto-drug antibodies.^8^ Consequently, routine use of therapeutic drug monitoring (TDM) paired with dose optimization before (proactive) or after (reactive) the development of gastrointestinal symptoms has been shown to improve rates of sustained steroid-free remission and intestinal healing.^7^^,^^9^^,^^10^ Additional strategies to optimize the effectiveness of the anti-TNF biologics in children include maximizing drug exposure during induction using higher doses (up to 10 mg/kg) or shortening the interval during maintenance to achieve higher concentration targets.^11^^,^^12^ For patients with adequate drug exposure and significant ongoing inflammation, however, the treatment strategy is to discontinue the anti-TNF and induce remission using an advanced therapy with an alternative mechanism of action.

The gold standard to evaluate treatment effectiveness remains invasive endoscopy. To minimize the use of repeat endoscopy, blood and fecal biomarkers are routinely used in real-world clinical practice to assess early and late responses to therapy. In clinical trials, endoscopic healing (EH) has traditionally been classified by the CD Endoscopic Severity Index^13^ or the simple endoscopic score-CD (SES-CD).^14^ In the recently published challenges in inflammatory bowel disease (IBD) Research 2024: Precision Medicine, the collaborative working group acknowledged a continued, urgent need for pharmacokinetic (PK) metrics and pharmacodynamic (PD) biomarkers “to predict disease course and response to therapies.”^15^ PK describes drug exposure over time, whereas PD captures the relationship between drug exposure and therapeutic effect. Ideally, PD biomarkers should be objective, correlate with intestinal injury, be specific to CD, and be easily obtainable.

The Fcγ receptor 1 (CD64) is a promising PD biomarker of endoscopic disease severity in CD.^16^ CD64 expression on neutrophils and monocytes is upregulated by interferon-γ and granulocyte-macrophage colony-stimulating factor during inflammation and primarily binds IgG1 and IgG3 facilitating cellular functions such as phagocytosis, antigen presentation and cytokine production.^17^ Previous studies have highlighted the clinical importance of neutrophil CD64, as it is upregulated during intestinal injury, associated with rapid infliximab clearance, and is associated with primary non-response to anti-TNF.^16^^,^^18^^,^^19^

In this study, our primary objective was to evaluate PK metrics and blood and stool PD biomarkers as surrogates for EH and to determine their optimal cut-points associated with EH as defined by the SES-CD. Our secondary objective was to use these PK and PD cut-points to develop a predictive model for EH.

## Materials and methods

### Study design and patient eligibility

In this multicenter, cross-sectional study, children and young adults (1-22 years old) with CD were approached for participation before undergoing a follow-up ileocolonoscopy. The study protocol was reviewed and approved by the Institutional Review Board (IRB) at all 6 participating centers, including Cincinnati Children’s Hospital Medical Center, Connecticut Children’s Medical Center, Nationwide Children’s Hospital, the Medical College of Wisconsin, Cleveland Clinic Children’s, and Dayton Children’s Hospital. Eligibility criteria included patients with CD receiving maintenance (>6 months) infliximab or adalimumab and scheduled for a clinically indicated colonoscopy between September 2019 and December 2020. As this was an observational study, all anti-TNF dosing regimens and timing of the endoscopy were at the discretion of the primary clinician. Patients with ulcerative colitis, inflammatory bowel disease-unspecified, or a known intestinal infection (including *Clostridium difficile*) in the previous 2 weeks before colonoscopy were excluded.

### Primary objective

The primary objective was to identify optimal cut-points for blood and stool PD biomarkers and PK metrics (drug concentration and clearance) for EH.

### Assessment of EH

The SES-CD was used to evaluate the endoscopic severity of the ileum and each colonic segment (cecum/ascending, transverse, descending/sigmoid, and rectum). The severity of affected intestinal segments is assessed based on the presence and size of the ulcerations and the presence of luminal narrowing or strictures.^20^ Each colonoscopy was scored by the primary endoscopist and then again by 2 separate reviewers (C.B. and P.M., if the endoscopy video was available) who were blinded to the indication of the procedure, the current therapeutic regimen, and the endoscopic report. EH was defined by a SES-CD < 3^21^ and the Cohen’s kappa was calculated to assess inter-rater agreement.

### PK analysis

At enrollment, the previous dose, interval, and date of the last infliximab (including any biosimilar) infusion and adalimumab injection were recorded. Infliximab, antibody to infliximab, adalimumab, and antibody to adalimumab concentrations were measured with a drug-tolerant electrochemiluminescence immunoassay (Esoterix, LabCorp Specialty Lab, Calabasas, CA). With serial dilutions, the infliximab assay had an upper detection limit of 600 μg/mL, a lower detection limit of 0.4 μg/mL, an inter-assay coefficient of variation (CV) of 6.07%-8.51% and a lower limit of antibody to infliximab detection of 22 ng/mL. The adalimumab assay had a lower detection limit of 0.6 µg/mL with an inter-assay CV of <10% and an antibody to adalimumab limit of detection of 25 ng/mL. As very few infliximab drug concentrations were collected as a trough, infliximab trough and clearance at steady state were estimated using the current dose (mg), the observed concentrations and the actual time after the last dose applying the Xiong et al. population PK model^19^ with Bayesian estimation using nonlinear mixed-effects modeling (NONMEM) software (version 7.5.1, ICON Development Solutions, Ellicott City, MD). To assess the reliability of the estimated infliximab trough concentrations derived using the Xiong et al. model, we used a second population PK model (developed by Fasanmade et al.^22^) and observed a strong correlation (*R*^2^ = 0.96) between the trough concentrations predicted by the 2 population PK models. Adalimumab trough concentration and clearance at steady-state were estimated using the observed concentrations and actual time after the last dose applying the Sharma et al. population PK model^23^ with Bayesian estimation. Further, to assess the reliability of the estimated adalimumab trough concentrations derived using the Sharma et al. model, we used a second population PK model (developed by Stodtmann et al.^24^) and observed a strong correlation (*R*^2^ = 0.99) between the concentrations predicted by the 2 population PK models.

### PD biomarker evaluation

Neutrophil surface expression of Fcγ Receptor I (CD64) was determined by a ratio of the mean fluorescence intensity (MFI) of granulocyte CD64 expression to the lymphocyte CD64 MFI by quantitative flow cytometry (FlowJo, BD Life Sciences), reported as the neutrophil CD64 activity ratio (nCD64) with the specific methods previously described.^16^ Monocyte surface expression of CD64 (mCD64) is determined from the same whole blood sample. Briefly, the expression of CD64 on monocytes is evaluated by labeling peripheral blood with CD64, CD163 (a monocyte marker) and CD45 (a pan-leukocyte marker) and analyzing the cells by flow cytometry (FlowJo). During analysis, patient leukocytes (lymphocytes, monocytes and granulocytes) are identified by their typical forward and side scatter with CD45 labeling. Monocytes are further identified with side scatter and CD163 labeling. The final mCD64 is reported as the ratio of monocyte CD64 MFI to the lymphocyte CD64 MFI. Human FCGRI/CD64 ELISA was performed according to the manufacturer’s manual (LifeSpan BioSciences, Inc., Seattle, WA) to determine the soluble CD64 (sCD64).^16^^,^^18^ Fecal calprotectin and fecal lactoferrin were determined from a stool sample collected >48 hours prior (up to 60 days prior) or >48 hours after the colonoscopy preparation using commercial kits (Buhlmann, Switzerland).

Additional PD biomarker testing (c-reactive protein [CRP], erythrocyte sedimentation rate [ESR], and platelet count and albumin) was performed at the discretion of the treating clinician with the results included in our study if the tests were collected up to 35 days before the endoscopy. Clinical remission (CR) was defined as a weighted pediatric CD activity index (wPCDAI) < 12.5 and off corticosteroids at the time of the endoscopy.^25^ Deep remission was defined as EH and CR.

### Statistical analysis

Continuous variables are represented as means with standard deviations (SD) or medians with 25%-75% interquartile range (IQR) depending on data distribution. Trough concentrations and drug clearance were compared between patients with EH and those without EH (non-EH) with either the Mann–Whitney test (nonparametric) or the Student’s *t*-test (parametric) based on the data distribution for each biomarker. Similarly, we assessed the differences in the PK and PD biomarkers for those who achieved deep remission and those with no deep remission. The receiver operating characteristic (ROC) curve was established for each PK metric and PD biomarker with the optimal cut-point (target) determined using Youden’s J statistic for each outcome of interest. For each cut-point, we also identified the area under the ROC curve (AUROC) and 95% confidence interval (CI) with sensitivity, specificity, positive predictive value (PPV) and the negative predictive value (NPV) calculated. Model comparisons were performed using DeLong’s test for correlated ROC curves.

We quantified the association between PD biomarkers and endoscopic scoring using Spearman’s rank correlation coefficients. We performed univariable logistic regression to assess the association between the biomarkers and EH, calculating odds ratios (ORs) and corresponding 95% CI. Following the univariate analysis, significant predictors (*P* < .05) were included in a multivariable regression model building unless the predictor had >15% missingness. We employed a penalized regression approach for variable selection and parameter estimation, combined with multiple imputation to handle missing data (R package “miselect”). Specifically, grouped adaptive least absolute shrinkage and selection operator (LASSO) was applied across 5 imputed data sets, where a group penalty was added to the aggregated objective function to ensure consistent feature selection across imputed data sets. We present model coefficients derived from a logistic regression of the raw data, due to over-conservative estimates provided by LASSO.

Statistical analyses were performed using GraphPad PRISM version 8.0.1 (GraphPad, Sand Diego, CA) and R (Core Team, 2020, version 4.4.1).

## Results

### Patient demographics by endoscopic outcome

In total, 91 patients were enrolled in the study. We excluded 4 patients, as 2 had an incomplete ileocolonoscopy and 2 did not have SES-CD scoring performed by the endoscopist. The remaining cohort of 87 participants was 48.3% female and 90.8% white, with a mean (SD) age of 16.3 (3.6) years. At the time of endoscopy, 62 (71.3%) of patients were receiving infliximab, with the entire cohort exposed to anti-TNF for a median (IQR) of 845 (385-1609) days. The primary indication for colonoscopy was to evaluate for EH (42.5%) or to evaluate the extent of inflammation (partial or no response to therapy) to support a decision to optimize anti-TNF dosing or a change in therapy class (57.5%).

EH was achieved in 48 patients (55.2%) in the entire cohort and 64.9% (24/37) of the patients referred for colonoscopy to assess EH, all of whom were in CR. There were no differences in gender, race, age at colonoscopy, or age at diagnosis between patients with non-EH or EH (Table 1). We found that patients on infliximab had a higher proportion of EH (62.9%) compared to adalimumab (36%, *P* = .03), while non-EH was seen in a higher rate of patients with the stricturing (B2) phenotype. There was no difference in EH between patients receiving combination therapy with an immunomodulator (*n* = 18; infliximab, *n* = 14, adalimumab, *n* = 4) and those receiving anti-TNF monotherapy (*n* = 69, *P* = .43). The intraclass correlation coefficient between the 2 blinded reviewers performing the SES-CD scoring was 0.91 (95% CI 0.84-0.95).

**Table 1:** Patient characteristics by endoscopic outcome.

Characteristic	Non-EH (*n* = 39)	Endoscopic healed (*n* = 48)	*P*-value
**Female, *n***	18 (46.2%)	24 (50%)	.83
**White race, *n***	33 (84.6%)	46 (95.8%)	.13
**Age at colonoscopy, years**	15.6 (3.5)	16.8 (3.6)	.11
**Age at diagnosis, years**	11.5 (3.7)	12.6 (3.5)	.14
**Type of anti-TNF**			
** Infliximab: adalimumab, *n***	23: 16	39: 9	.03
**Time on anti-TNF, days**	1038 (771)	1145 (896)	.46
**Receiving combination therapy, *n***	10 (25.6%)	8 (16.7%)	.43
** Infliximab** **+** **methotrexate, *n***	4	4	–
** Infliximab** **+** **6-mercaptopurine/azathioprine, *n***	3	2	–
** Adalimumab** **+** **methotrexate, *n***	2	2	–
** Adalimumab** **+** **6-mercaptopurine, *n***	1	0	–
**Crohn’s location**			
** Ileal only: Colon only: Ileocolonic, *n***	9:6:24	12:9:27	–
**Crohn’s behavior**			
** Inflammatory, *n***	22 (56.4%)	37 (77.1%)	.06
** Stricturing, *n***	15 (38.5%)	6 (12.5%)	.006
** Penetrating, *n***	1 (2.6%)	3 (6.3%)	.63
** Both, *n***	1 (2.6%)	2 (4.2%)	.99
**Perianal fistula, *n***	9 (23%)	8 (16.7%)	.59
**Indication for colonoscopy**			
** Evaluate for mucosal healing**	13 (33.3%)	24 (50%)	.13
** Evaluate extent of injury (partial or no) response**	26 (66.7%)	24 (50%)	–

Non-EH, not endoscopic healed with a simple endoscopic score-Crohn’s disease ≥ 3. Data are presented as mean (standard deviation) or as a number (*n*) with a percentile. Abbreviations: EH, endoscopic healing; TNF, tumor necrosis factor-alpha.

### Clinical disease activity is not associated with EH

We found the wPCDAI scores did not significantly differ between EH and non-EH, underlining the ongoing need to identify objective biomarkers of active bowel inflammation. In fact, 40.6% of those with a wPCDAI < 12.5 were found to have non-EH (Table 2).

**Table 2:** Cut-points for several peripheral blood and stool pharmacodynamic biomarkers associated with endoscopic healing.

Biomarker	Non-EH	Endoscopic healed	Youden Cut-point	AUROC (95% CI)	*P*-value
**Clinical activity score**					
**Weighted pediatric Crohn’s disease activity index**	7.5 (0-20)	0 (0-10)	10	0.61 (0.49-0.72)	.068
**Routine biomarkers**					
**Platelet count, ×10^3^/µL**	316 (249-382)	245 (222-277)	294	0.78 (0.67-089)	<.001
**Serum albumin, g/dL**	3.9 (3.5-4.2)	4.2 (4-4.4)	3.9	0.76 (0.64-0.87)	<.001
**Erythrocyte sedimentation rate, mm/h**	9 (6-18)	6 (5-10)	15	0.63 (0.49-0.77)	.074
**C-reactive protein, mg/dL**	0.5 (0.3-0.82)	0.29 (0.09-0.4)	0.5	0.75 (0.63-0.87)	<.001
**Vitamin D 25-hydroxy, ng/mL**	28.4 (26.8-35.3)	35 (32-40)	31	0.70 (0.5-0.91)	.065
**Fecal calprotectin, µg/g**	879 (318-1428)	95 (30-263)	402	0.86 (0.76-0.96)	<.001
**Exploratory biomarkers**					
**Soluble CD64, ng/mL^a^**	19.7 (7)	15.9 (5.9)	15.8	0.67 (0.54-0.79)	.014
**Neutrophil CD64, ratio**	5.03 (3.9-6)	3.7 (3.3-4.3)	4.5	0.76 (0.64-0.88)	<.001
**Monocyte CD64, ratio**	44.6 (33.5-52.1)	37 (31.1-43.3)	44.6	0.67 (0.52-0.82)	.023
**Fecal lactoferrin, µg/g**	23.6 (4.2-60.4)	3.7 (2.6-14.2)	23.6	0.78 (0.64-0.92)	.0013

Non-EH, not endoscopic healed, simple endoscopic score-Crohn’s Disease ≥ 3. Biomarkers are presented as median (25%-75%) or ^*a*^mean (±SD) based on data distribution. Abbreviations: AUROC, Area under receiver operating characteristic; CI, confidence interval; EH, endoscopic healing; TNF, tumor necrosis factor-alpha.

### PD biomarkers associated with EH

Next, we assessed the differences in routine and more novel laboratory markers between non-EH and EH. We found there were significant differences in the platelet count, serum albumin, CRP, and fecal calprotectin (Table 2). While the median (IQR) CRP was significantly higher in non-EH as compared to EH, only 32.3% of the non-EH cohort had an elevated CRP (>0.5 mg/dL) while 89.3% of the non-EH cohort had an elevated fecal calprotectin (>150 µg/g).

**Figure 1:**
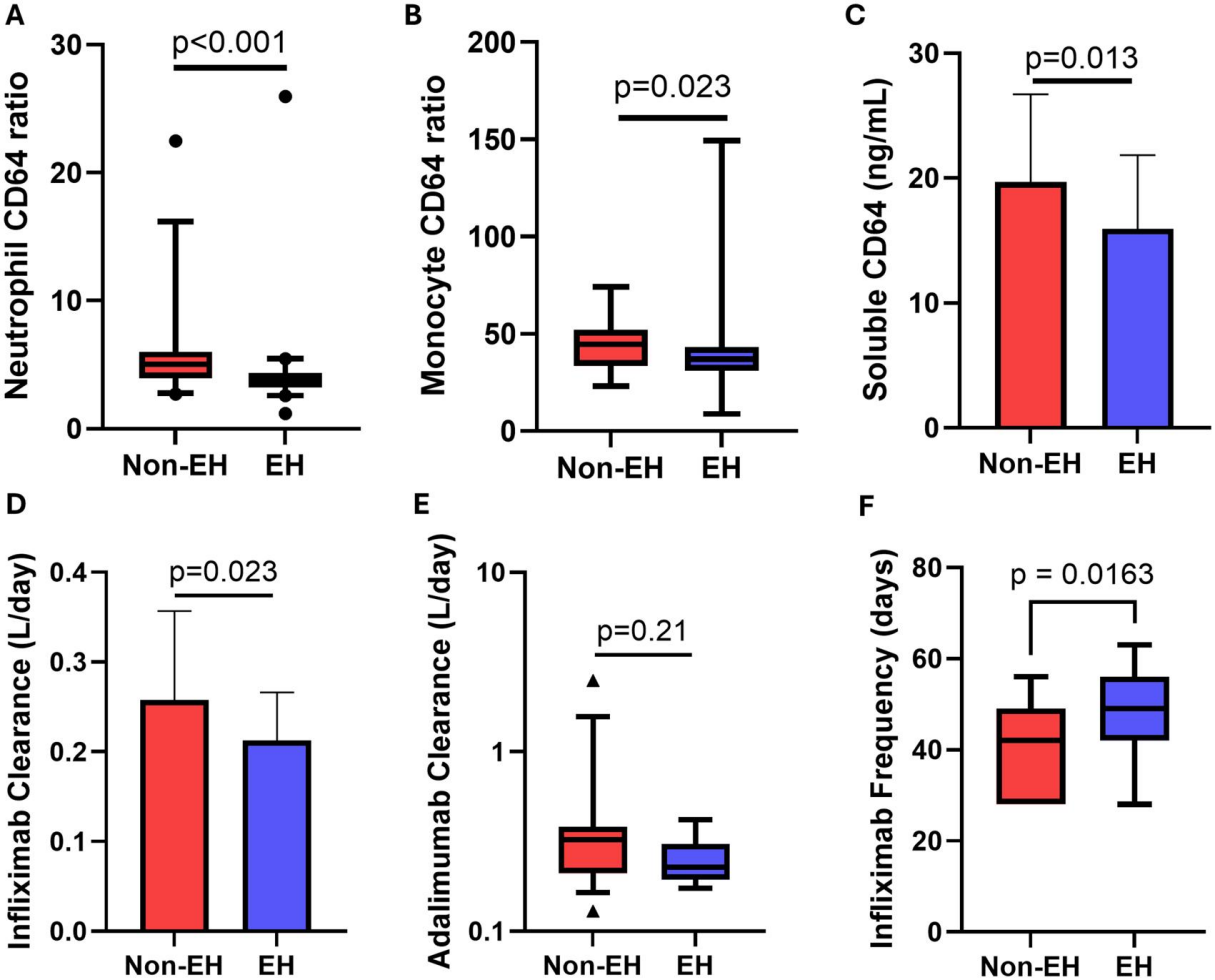
Novel pharmacodynamic and pharmacokinetic biomarkers for endoscopic activity. Non-endoscopic healed (non-EH) was defined by a simple endoscopic score-Crohn’s disease (CD) ≥ 3. We found (A) the neutrophil CD64 ratio, (B) the monocyte CD64 ratio, and (C) the soluble CD64 were elevated in non-EH compared to those who achieved EH. (D) Infliximab clearance was found to be increased in non-EH versus EH while there was no difference in (E) adalimumab clearance. (F) Patients on infliximab with EH had a longer dosing frequency (days) compared to non-EH. Infliximab clearance at the time of the endoscopy was estimated using the Xiong et al.^19^ population pharmacokinetic model and the available covariates. Adalimumab clearance at the time of the endoscopy was estimated using the Sharma et al.^23^ population pharmacokinetic model and available covariates.

**Figure 2:**
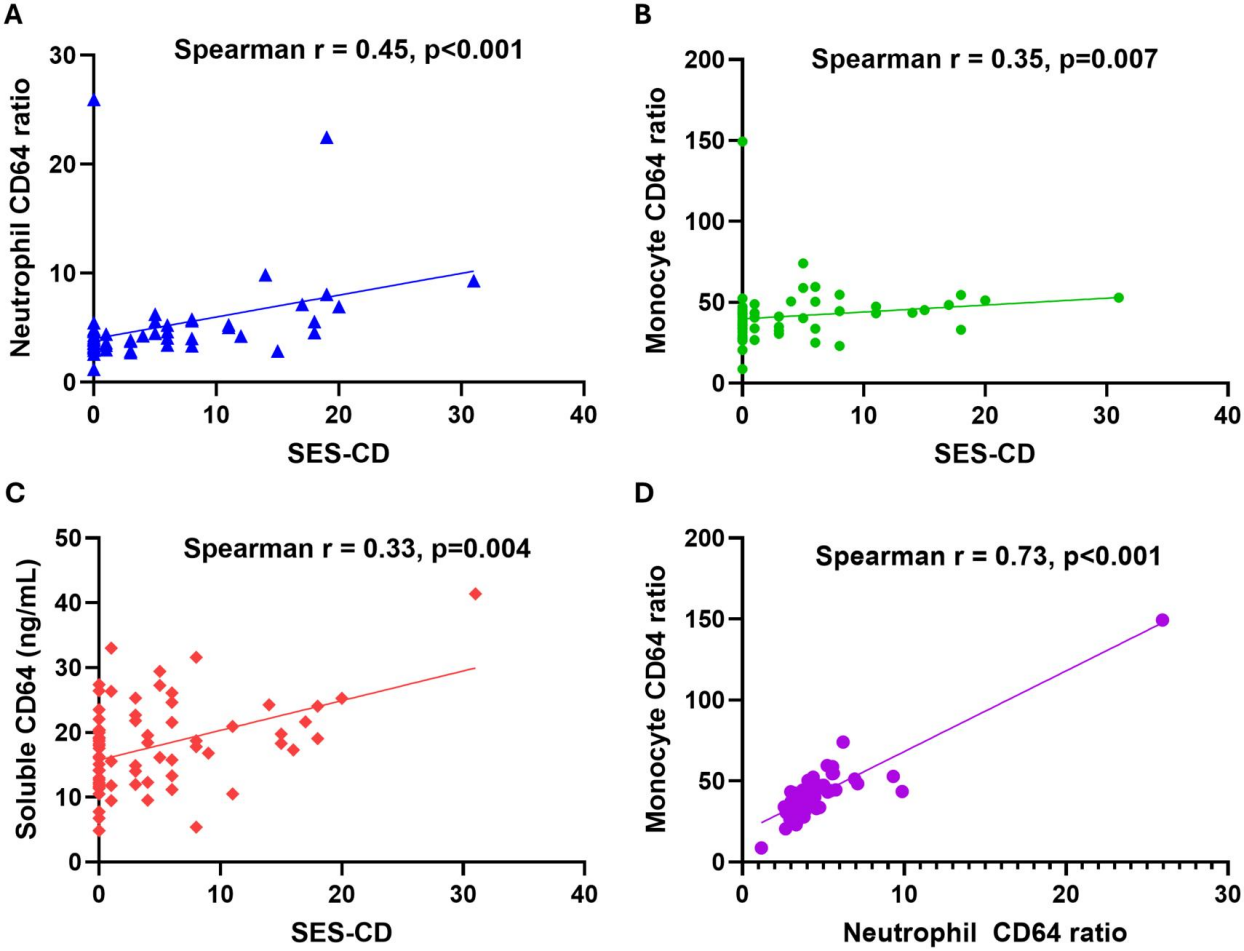
The novel CD64 biomarkers demonstrated good correlation with endoscopic severity. The simple endoscopic severity-Crohn’s disease (SES-CD) score was compared to (A) the neutrophil CD64 ratio, (B) the monocyte CD64 ratio, and (C) the soluble CD64 concentration at the time of the colonoscopy. (D) There was a strong correlation between neutrophil CD64 ratio and the monocyte CD64 ratio.

We also assessed whether the novel peripheral blood biomarkers nCD64, mCD64, and sCD64 were associated with EH (Table 2). We found the nCD64, mCD64 and sCD64 were significantly elevated in non-EH with a median (IQR) ratio of 5.03 (3.9-6), ratio of 44.6 (33.5-52.1), and a mean (SD) of 19.7 ng/mL (7), respectively (Figure 1A–C). The ideal cut-points for non-EH were identified for each biomarker. We found the specificity for EH was 82.9% for nCD64 (<4.5 ratio), 85.3% (<44.6 ratio) for mCD64, and 56.1% for sCD64 (<15.8 ng/mL, Figure S1). We used the Spearman correlation to determine the relationship between CD64 and SES-CD scores. While not as strong of a correlation as fecal calprotectin (*r* = 0.7, *P* < .001), nCD64 had a moderate overall correlation with the SES-CD (*r* = 0.45, *P* < .001, Figure 2A). The correlations between the SES-CD scores with mCD64 and sCD64 were less robust (*r* = 0.35 and *r* = 0.33, respectively, Figure 2B, C). Finally, we found the mCD64 ratio strongly correlated with the nCD64 ratio (*r* = 0.73, *P* < .001, Figure 2D).

### PK biomarkers and endoscopic outcomes

We next assessed the association between anti-TNF PK metrics in EH and non-EH. In this contemporary cohort, there were no differences in the predicted infliximab or adalimumab trough concentrations between EH and non-EH (Table 3). However, patients with non-EH had more rapid drug clearance of infliximab with a mean (SD) rate of 0.26 (0.1) L/day compared to 0.21 (0.05) L/day in patients with EH (*P* = .023, Figure 1D). While adalimumab clearance did not reach statistical significance in the cohort, patients with EH had a median clearance of 0.23 (0.19-0.31) L/day compared to 0.32 (0.21-0.38) L/day in patients with non-EH (*P* = .21, Figure 1E). Infliximab frequency was significantly increased in patients with non-EH with a median (IQR) of every 42 days (28-49) compared to every 49 days (42-56) for patients with EH (Figure 1F).

**Table 3:** Anti-TNF dosing regimens and pharmacokinetic metrics between non-endoscopic healed and endoscopic healed at the time of the colonoscopy.

Characteristic	Non-EH	Endoscopic healed	*P*-value
**Currently receiving infliximab, *n***	23	39	
**Infliximab dose, mg/kg^a^**	9.2 (1.9)	8.3 (2.5)	.10
**Infliximab frequency, days**	42 (28-49)	49 (42-56)	.016
**Infliximab predicted trough, µg/mL**	10.1 (5.4-14.6)	11.2 (8.5-16.2)	.38
**Infliximab clearance, L/day^a^**	0.26 (0.10)	0.21 (0.05)	.023
**Currently receiving adalimumab, *n***	16	9	
**Adalimumab dose, mg/kg^a^**	0.70 (0.27)	0.74 (0.27)	.69
**Adalimumab frequency, days**	12 (7-14)	14 (7-14)	.99
**Adalimumab predicted trough, µg/mL**	7.5 (5.6-12.7)	8.9 (6.2-12.3)	.55
**Adalimumab clearance, L/day**	0.32 (0.21-0.38)	0.23 (0.19-0.31)	.21

Infliximab trough in the maintenance phase was predicted using the Xiong et al. population pharmacokinetic model and Bayesian estimation while adalimumab maintenance trough was predicted using the Sharma et al. population pharmacokinetic model and Bayesian estimation. Biomarkers are presented as median (25%-75%) or ^*a*^mean (±SD) based on data distribution. Abbreviation: EH, endoscopic healing.

### PD and PK biomarkers associated with deep remission

These biomarkers were also evaluated in the 43.7% of patients in the cohort who were in deep remission (CR and EH). We found the platelet count, CRP, sCD64, nCD64 ratio, fecal calprotectin, and fecal lactoferrin were all significantly lower in patients who achieved deep remission compared to those who did not (Table S1). While some biomarker cut-points for EH and deep remission were similar, there were notable differences in the cut-points for sCD64, fecal calprotectin and fecal lactoferrin. While not statistically significant, we found there was a trend for more rapid drug clearance for both infliximab (*P* = .059) and adalimumab (*P* = .057) for those who were not in deep remission.

### Univariate predictors for EH

As noted, PK metrics and PD biomarker cut-points were established for EH with an ROC analysis and the Youden Index (Table 2). The cut-points for each biomarker were then used to identify OR in our univariate regression analysis (Table 4). From this analysis, the novel biomarkers sCD64 (OR 3.7, 95% CI: 1.4-10.2), nCD64 ratio (OR 8, 95% CI: 2.7-25.5) and mCD64 ratio (OR 6.3, 95% CI 1.9-23.4) were all found to be associated with EH. While the identified cut-point of <402 µg/g for fecal calprotectin was strongly associated with EH (OR 17.3, 95% CI: 4.8-76.5), we also evaluated a fecal calprotectin of <250 µg/g (13.1, 95% CI: 3.9-52.9), as this cut-point is well established for EH. Interestingly, infliximab clearance rate <0.23 L/day was not associated with deep remission, but we found that adalimumab clearance rate <0.29 L/day was associated with deep remission (OR 15.4, 95% CI: 2-332, Table S2).

**Table 4:** Univariate analysis to assess predictors for endoscopic healing.

Predictors for endoscopic healing		
Predictor	Odds ratio (95% CI)	*P*-value
**On infliximab therapy**	3 (1.2-8.2)	.025
**On an immunomodulator**	0.58 (0.2-1.7)	.31
**Infliximab clearance <0.23 L/day**	3.2 (1.1-9.7)	.04
**Adalimumab clearance <0.29 L/day**	7.7 (1.3-66.2)	.035
**Platelet count <294** **000**	15.8 (4.9-63)	<.001
**Albumin ≥3.9 g/dL**	12.2 (3.5-58)	<.001
**C-reactive protein <0.5 g/dL**	6.7 (2.3-22)	<.001
**Soluble CD64 < 15.8 ng/mL**	3.7 (1.4-10.2)	.009
**Neutrophil CD64 < 4.5 ratio**	8 (2.7-25.5)	<.001
**Monocyte CD64 < 44.6 ratio**	6.3 (1.9-23.4)	.003
**Fecal calprotectin <402 µg/g**	17.3 (4.8-76.5)	<.001
**Fecal calprotectin <250 µg/g**	13.1 (3.9-52.9)	<.001

Abbreviation: CI, confidence interval.

### Multivariable LASSO regression to predict EH

**Figure 3:**
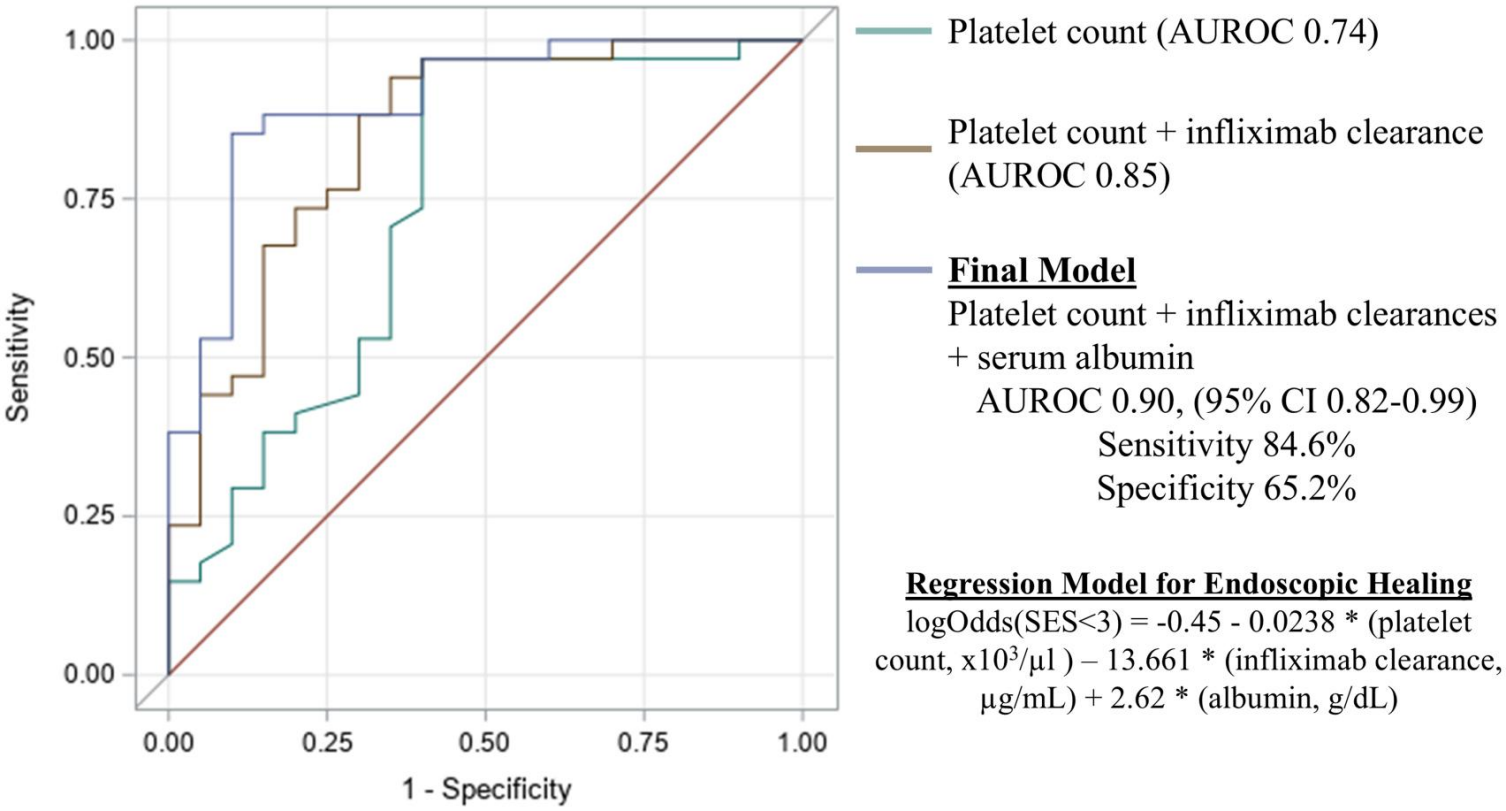
Three routine biomarkers are strongly associated with endoscopic healing. The univariate analysis identified several pharmacodynamic biomarkers and pharmacokinetic metrics associated with endoscopic healing (EH). We performed a multivariable regression analysis from the significant predictors (biomarkers) with <15% missingness. The addition of the platelet count, infliximab clearance, and serum albumin led to a final model with an area under the receiver operator characteristic (AUROC) curve of 0.9 when all 3 predictors were used in the model. CI, confidence interval.

To utilize these biomarkers in a predictive model, we first imputed missing data to generate 5 imputed data sets. The analysis was limited to patients receiving infliximab only due to a smaller number of patients receiving adalimumab (28.7% of the cohort). LASSO regression was used across each of the 5 data sets to select biomarkers for inclusion in a predictive model for EH. Biomarkers with >15% missingness were left out of data imputation and the model build which included nCD64 (19.6%), mCD64 (32.8%), fecal lactoferrin (50.6%), and fecal calprotectin (36.8%). To establish a baseline model, we built a model using CRP alone, which yielded an AUROC of 0.75 (95% CI 0.62-0.89). The final model, which included infliximab clearance, serum albumin, and platelet count, achieved a significantly higher AUROC of 0.90 (95% CI 0.82-0.99, Figure 3) compared to the CRP-only model (*P* = .02). Using a per-subject probability cutoff for having an SES-CD < 3 as a probability of >0.3, the sensitivity was 84.6% (69.5%-94%) and specificity was 65.2% (42.7%-83.6%) with a PPV of 80.5% (69.9%-88%) and NPV of 71.4% (53.0%-84.7%).

## Discussion

Identifying novel biomarkers and their respective cut-points for ongoing gut inflammation is critical to utilizing PK/PD monitoring in real-world clinical practice. Here, we identified cut-points for PK metrics, infliximab and adalimumab clearance and for PD biomarkers, platelet count, serum albumin, CRP, fecal calprotectin, and the novel CD64 markers, that were associated with EH in patients with CD treated with either infliximab or adalimumab.

As recently reviewed by Ananthakrishnan et al., biomarkers are used frequently for evaluation of EH and monitoring anti-TNF response in real-world practice.^21^ To date, fecal calprotectin, is the most sensitive biomarker of gut inflammation and has been used in conjunction with infliximab or adalimumab trough concentrations to predict loss of response in adult patients.^26^ Despite the high sensitivity for gut inflammation, the specificity for EH is relatively low (62.2%) when a cut-point of 250 µg/g was used.^27^ Other general inflammatory markers, including CRP and ESR, are also frequently used in the clinical setting to monitor disease activity.^28^ While elevations in these inflammatory markers are associated with disease activity, the sensitivities are relatively poor, and cut-points are not well-established for pediatric patients with non-EH, making it difficult to interpret lab values. Using the Youden index, we identified cut-points for EH for the traditional PD biomarkers, including CRP, platelet count, serum albumin, and fecal calprotectin. More importantly, we found 40.6% of the cohort with non-EH had a normal wPCDAI score further highlighting the difficulty in evaluating gut inflammation when relying only on subjective disease activity scores.

In children with CD, several real-world studies and 1 clinical trial has demonstrated that anti-TNF dose optimizations following TDM is a key intervention to improve the durability of the anti-TNF biologic.^7^^,^^10^^,^^29^ In clinical practice, anti-TNF dosing is often optimized following PD (blood, stool, imaging, and/or colonoscopy) or PK monitoring. Before the more routine practice of using proactive TDM to ensure adequate exposure before performing a repeat colonoscopy, investigators were able to show a clear association between anti-TNF drug concentrations and the rate of mucosal healing.^30^ Surprisingly, we did not find any difference in the median drug concentrations between EH and non-EH. We expect this finding was related to the change in practice with the clinician dose optimizing the anti-TNF biologic before scheduling the colonoscopy, as the participating centers trial optimal drug exposure before stopping anti-TNF therapy. Although anti-TNF concentrations were estimated values and not true trough concentrations, we reported the reliability of these estimations with separate models for each biologic (noted in the Methods) and observed a strong correlation between the trough concentrations for infliximab (*R*^2^ = 0.96) and adalimumab (*R*^2^ = 0.99).^18^^,^^19^ We did find, however, that increased infliximab clearance was associated with non-EH. While we did not find a statistical difference in the median adalimumab clearance between non-EH and EH, we did find that patients with adalimumab clearance of <0.29 L/day (the ideal cut-point) were more likely to have achieved EH and deep remission. These findings strongly suggest that both drug concentration and drug clearance should be evaluated when assessing treatment failure. For instance, the discovery of rapid drug clearance may suggest a role for further dose optimization or the use of combination therapy to improve the inflammatory burden.

Along with more routine PD biomarkers, we also investigated the utility of the novel biomarker, CD64. Identifying an association between endoscopic inflammation with elevated nCD64, mCD64, and sCD64 not only provides a robust peripheral blood biomarker to follow throughout the disease course, but may also serve as a companion diagnostic for advanced therapies, such as the IL-23 inhibitor guselkumab, that can selectively bind to CD64.^31^ The guselkumab monoclonal antibody differs from other IL-23 inhibitors in that it is a fully human IgG1 monoclonal antibody with a native Fc region. CD64 has been shown to be upregulated on the surface of IL-23-producing monocytes and can bind to the Fc fragment of guselkumab, which may help anchor guselkumab in a position to neutralize IL-23 as it is released from CD64^+^ monocytes.^31^ While nCD64 and mCD64 were not included in the final multivariable regression model due to missingness, future larger studies are currently being conducted to further validate the strong associations we discovered during our univariate analysis.

While larger validation studies in both children and adults are needed, our study is highly relevant to clinicians as the American Gastroenterological Association (AGA) recently published clinical practice guidelines for biomarker monitoring in adult patients with CD. The AGA recommended a monitoring strategy that combines the assessment of both biomarkers and gastrointestinal symptoms, rather than relying on symptoms alone with interval biomarker monitoring to be performed every 6-12 months for adults with CD in CR.^21^ While they summarize that additional data is required for the monitoring schedule of endoscopy in patients with CD, the AGA made no recommendation in favor of, or against, a biomarker-based monitoring strategy over an endoscopy-based monitoring strategy to improve long-term outcomes. Our discovery biomarker data suggests that EH would be more likely for a patient receiving infliximab that has a platelet count <308 000, an albumin >4 g/dL and an infliximab clearance <0.23 L/day. We acknowledge that the assessment of drug clearance is complex and requires a software tool for Bayesian forecasting using a population PK model. However, as PK dashboards and software tools become more widely available,^32^ future studies will be needed to evaluate outcomes when both drug clearance and drug concentrations are used to assess response and optimize biologic dosing. Moreover, our data analysis focused on EH to align with current STRIDE II guidelines, as the impact of histological healing on long-term outcomes in CD has not yet been established.^33^ Finally, larger cohorts are needed to determine the role of measuring nCD64 and mCD64 at key intervals (induction and immediately post-induction) during treatment and the yield on capturing multiple, longitudinal assessments of these biomarkers throughout treatment.

Strengths of our study include the use of a multicenter cohort design, the use of PK modeling to predict drug trough concentrations, estimating drug clearance at the time of endoscopy, and establishing cut-points for the PK metrics and PD biomarkers using a well-established endoscopic severity score to assess EH. Furthermore, to our knowledge, this is the first investigation to find an association between increased mCD64 and endoscopic severity scores. Limitations of our study include the relatively small sample size, a high proportion of patients receiving infliximab compared to adalimumab, use of estimated rather than directly measured drug concentrations due to blood samples collected at the time of endoscopy, and missingness of data for several biomarkers like fecal calprotectin. As we identified that high infliximab clearance was associated with non-EH and an elevated adalimumab clearance ≥0.29 L/day was seen with patients failing to achieve EH, we suspect that a larger population of children receiving adalimumab would have provided more robust data to develop a multivariable model for adalimumab and EH.

In conclusion, we identified cut-points for routine PD biomarkers associated with EH, as well as cut-points for the novel CD64 biomarkers. Furthermore, we found increased infliximab clearance, but not drug trough concentrations, was associated with endoscopic outcomes. Taken together, our findings will help identify patients with PK and/or PD failure and, eventually, the development of a more personalized approach to the management of patients receiving anti-TNF biologics. Future directions will include an external validation of our model using a larger patient cohort and evaluating longitudinal assessments of the CD64 tests as biomarkers of sustained healing. We envision the real-world use of these peripheral blood biomarkers to develop a more systematic approach to assessing and responding to PD non-response and a more proactive process for the early identification of silent CD before overt treatment failure.

## Supplementary Material

otag088_Supplementary_Data

## Data Availability

Once published, the de-identified dataset will be made available to researchers upon request to the corresponding author.

## Conference presentation

Part of the data was presented as an oral presentation at the NASPGHAN Annual Meeting 2022 during the Single Topic Symposium in Orlando, Florida, and as an oral presentation at the 2025 Crohn’s and Colitis Congress in San Francisco, CA.
