# Supplementary material for: Evaluation of pharmacokinetic metrics and pharmacodynamic biomarkers associated with endoscopic healing in pediatric Crohn’s disease patients treated with anti-tumor necrosis factor biologics
Source: Crohns Colitis 360. 2026 Jul 27;8(3):otag088. doi: 10.1093/crocol/otag088 (PMC13457079; doi:10.1093/crocol/otag088)
Supplement: Supplementary file 1 [file otag088_supplementary_data.docx]

**Supplemental Materials**

**Supplemental Table 1. Pharmacokinetic and Pharmacodynamic Biomarkers in Patients with Deep Remission**

| **Characteristic** | **No Deep Remission (n=49)** | **Deep**  **Remission**  **(n=38)** | **Youden Cut-point** | **AUROC**  **(95% CI)** | **p-value** |
| --- | --- | --- | --- | --- | --- |
| **Blood Pharmacodynamic Biomarkers** | | | | | |
| Platelet count, x10^3^/µl | 311 (237-370) | 250 (223-277) | 311 | 0.68 (0.55-0.80) | 0.008 |
| Serum albumin, g/dL | 4 (3.6-4.3) | 4.2 (4-4.4) | 3.9 | 0.67 (0.55-0.79) | 0.011 |
| C-reactive protein, mg/dL | 0.4 (0.19-0.78) | 0.29 (0.14-0.4) | 0.5 | 0.66 (0.53-0.78) | 0.025 |
| Soluble CD64, ng/mL*^a^* | 19 (7.3) | 15.6 (5.2) | 20.3 | 0.64 (0.52-0.77) | 0.028 |
| Neutrophil CD64, ratio | 4.4 (3.4-5.6) | 3.8 (3.1-4.4) | 5 | 0.68 (0.56-0.81) | 0.008 |
| Monocyte CD64, ratio | 41.2 (33.2-51) | 38.7 (33.2-43.6) | 47.4 | 0.58 (0.43-0.73) | 0.32 |
| **Fecal Pharmacodynamic Biomarkers** | | | | | |
| Fecal calprotectin, µg/g | 740 (241-1175) | 93 (30-263) | 195 | 0.83 (0.73-0.94) | <0.001 |
| Fecal lactoferrin, µg/g | 16.5 (3.9-57) | 3.7 (2.1-19.6) | 3.8 | 0.72 (0.57-0.88) | 0.012 |
| **Pharmacokinetic Biomarkers** | | | | | |
| Infliximab trough, µg/mL | 10 (8.7-14.9) | 11.6 (7.4-14.4) | 12 | 0.52 (0.37-0.66) | 0.84 |
| Infliximab clearance, L/day*^a^* | 0.25 (0.09) | 0.21 (0.06) | 0.23 | 0.61 (0.47-0.75) | 0.059 |
| Adalimumab trough, µg/mL | 7.5 (5.6-11.6) | 9.4 (6.8-12.8) | 8.9 | 0.63 (0.39-0.86) | 0.34 |
| Adalimumab clearance, L/day | 0.33 (0.23-0.39) | 0.22 (0.19-0.28) | 0.29 | 0.74 (0.54-0.94) | 0.057 |

Deep Remission is defined by simple endoscopic score-Crohn’s disease <3 and a weighted pediatric Crohn’s disease activity index <12.5. AUROC, area under the receiver operating characteristic curve; CI, confidence interval. Biomarkers are presented as median (25-75%) or *^a^*mean (±SD) based on data distribution.

**Supplemental Table 2. Univariate Analysis to Assess Predictors for Deep Remission**

| **Predictor** | **Odds Ratio**  **(95% CI)** | **p-value** |
| --- | --- | --- |
| On infliximab therapy | 2 (0.77-5.5) | 0.17 |
| On combination therapy | 0.58 (0.18-1.7) | 0.32 |
| Infliximab clearance <0.23 L/day | 2.9 (0.99-9.1) | 0.057 |
| Adalimumab clearance <0.29 L/day | 16.8 (2.2-361) | 0.018 |
| Platelet count >311,000 | 10.1 (3-47.2) | <0.001 |
| Albumin ≥3.9 g/dL | 7 (2-32.6) | 0.005 |
| C-reactive protein <0.5 g/dL | 4.9 (1.6-16.8) | 0.007 |
| Soluble CD64 <20.3 ng/mL | 4.9 (1.6-18.8) | 0.0095 |
| Neutrophil CD64 <5 | 6.8 (2-32.1) | 0.006 |
| Monocyte CD64 <47.4 | 6.9 (1.6-47.4) | 0.019 |
| Fecal calprotectin <195 µg/g | 15.3 (4.3-64) | <0.001 |
| Fecal calprotectin <250 µg/g | 8.5 (2.6-31) | <0.001 |
| Fecal lactoferrin <3.8 µg/g | 4 (1.1-16.5) | 0.044 |

Deep Remission is defined by simple endoscopic score-Crohn’s disease <3 and a weighted pediatric Crohn’s disease activity index <12.5.

**Supplemental Figure 1. Novel CD64 Biomarkers are Associated with Endoscopic Healing.** The receiver operating curve (ROC) was established for (a) neutrophil CD64 ratio, (b) monocyte CD64 ratio, and (c) soluble CD64. Optimal cut-points were determined using Youden’s J statistic for each biomarker. Sensitivity, specificity, positive predictive value (PPV), and negative predictive value (NPV) were also calculated for each CD64 biomarker.
